# Dielectric Relaxation of La-Doped Zirconia Caused by Annealing Ambient

**DOI:** 10.1007/s11671-010-9782-z

**Published:** 2010-09-30

**Authors:** C Z Zhao, M Werner, S Taylor, P R Chalker, A C Jones, Chun Zhao

**Affiliations:** 1 Department of Electrical and Electronic Engineering, Xi'an Jiaotong, Liverpool University, 215123, Suzhou, Jiangsu, China; 2 Department of Electrical Engineering and Electronics, University of Liverpool, Liverpool, L69 3GJ, UK; 3 Department of Engineering, Materials Science and Engineering, University of Liverpool, Liverpool, L69 3GH, UK; 4 Department of Chemistry, University of Liverpool, Liverpool, L69 3ZD, UK

**Keywords:** La, ZrO_2_, La_0.35_Zr_0.65_O_2_

## Abstract

La-doped zirconia films, deposited by ALD at 300°C, were found to be amorphous with dielectric constants (k-values) up to 19. A tetragonal or cubic phase was induced by post-deposition annealing (PDA) at 900°C in both nitrogen and air. Higher k-values (~32) were measured following PDA in air, but not after PDA in nitrogen. However, a significant dielectric relaxation was observed in the air-annealed film, and this is attributed to the formation of nano-crystallites. The relaxation behavior was modeled using the Curie–von Schweidler (CS) and Havriliak–Negami (HN) relationships. The k-value of the as-deposited films clearly shows a mixed CS and HN dependence on frequency. The CS dependence vanished after annealing in air, while the HN dependence disappeared after annealing in nitrogen.

## Introduction

Amorphous ZrO_2_ is one of the most promising dielectrics (dielectric constant k-value ~20) to replace SiO_2_ in MOSFETs at the 45-nm node CMOS technologies. Due to the aggressive down-scaling of MOSFET, higher dielectric constant materials and higher mobility semiconductors other than silicon are introduced [1-11]. Germanium is considered to be a good candidate to replace silicon in the channel of next-generation high-performance CMOS devices, while rare earth oxides belonging to another class of materials offer good passivation of germanium to reduce the density of interface states, as it has recently been suggested [5,7,10]. On the other hand, theoretical studies have reported that the metastable tetragonal and cubic phases (t- and c-phases) of ZrO_2_ have higher *k*-values [12,13]. The addition of rare earth elements, such as La, Gd, Dy, or Er, is reported to stabilize these phases and k-values of up to 40 have been obtained [7-11,14].

In order to induce the t- and c-phases in the La-doped ZrO_2_, dielectric post-deposition annealing (PDA) is needed, otherwise the layers grown by atomic layer deposition (ALD) at relatively low temperatures (<450°C) have an amorphous microstructure [15,16]. However, the transformation from amorphous to t- and c-phases can cause both dielectric relaxation and an adverse increase in the leakage current [14,17]. Leakage, which is the quantity defined in the ITRS Roadmap, depends on the combination of k-value and energy offset values between the energy bands of the high-*k* material and the silicon crystal. For example, 1 × 10^-8^ A/cm^2^ is a value required for DRAM capacitors [18] (much higher values are accepted for gate oxides in CMOS). Since the purpose to introduce high-k dielectrics is to reduce the leakage current of gate oxides, a lot of investigations on the leakage current of high-k dielectrics have been carried out [19-23].

However, there is little information about dielectric relaxation of La-doped ZrO_2_ dielectrics. Since loss due to the dielectric relaxation can cause MOSFET deterioration, the aim in this study was therefore to investigate the effect of PDA on the relaxation behavior of La-doped ZrO_2_. In this paper, we report the influence of the annealing ambient on the dielectric relaxation processes, which can be described by both the Havriliak–Negami (HN) and Curie–von Schweidler (CS) relationships [24-27] in the frequency range of 10 MHz.

## Experimental

La-doped ZrO_2_ films, with a thickness of 35 nm, were deposited on n-type Si(100) substrates by liquid injection ALD at 300°C, using a modified Aixtron AIX 200FE AVD reactor configured for liquid injection [28]. Both Zr and La sources are Cp-based precursors ([(MeCp)_2_ZrMe(OMe)] and [(^i^PrCp)_3_La]) [15,16]. The composition of the La-doped ZrO_2_ films was estimated to be La_0.35_Zr_0.65_O_2_ from Auger electron spectroscopy (AES). Selected films were annealed at 700°C or 900°C for 15 min, in an N_2_ or air ambient.

The effects of PDA on the physical and electrical properties of the La_0.35_Zr_0.65_O_2_ films have been investigated using cross-section transmission electron microscopy (XTEM), X-ray diffraction (XRD), high–low frequency capacitance–voltage (C–V), capacitance–frequency (C–f), and current–voltage (I–V) measurements, respectively.

In order to perform the C–V, C–f and I–V measurements, metal (Au) gate electrodes were evaporated to form metal–oxide–semiconductor capacitors (Au/La_0.35_Zr_0.65_O_2_/IL/n-Si, where IL stands for interfacial layer) with an effective contact area of 4.9 × 10^-4^ cm^2^. The backside of the Si wafer was cleaned with a buffered HF solution, and subsequently a 200-nm-thick film of Al was deposited to form an ohmic back contact. A thermal SiO_2_ sample was grown using dry oxidation at 1100°C to provide a comparison with the high-k stacks. Its back-side contact was prepared in exactly the same way as for all other La_0.35_Zr_0.65_O_2_ samples: depositing Al after HF treatment.

## Results and Discussion

**Figure 1:**
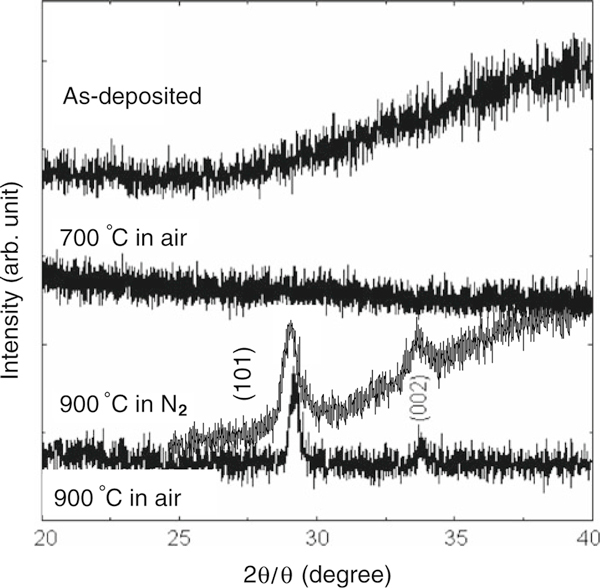
**X-ray diffraction data for La_0.35_Zr_0.65_O_2_ films deposited by ALD and then annealed in air or N_2_ for 15 min at different temperatures**.

XRD was carried out using a Rikagu Miniflex X-ray diffractometer with nickel-filtered Cu Kα radiation (λ = 1.5405 Å) and a 2θ increment of 0.2° per minute, and the results are shown in Figure 1. Results from the as-deposited samples and samples annealed at 700°C showed that the films were amorphous. XRD spectra from both samples annealed at 900°C show two clear diffraction peaks at 29.3° and 33.9°, suggesting that crystallization starts between 700 and 900°C. These peaks correspond to the t- or c- phases, but it is difficult to distinguish between them. Selected area diffraction results (not shown) obtained using a TEM would suggest that the cubic phase is the most likely.

**Figure 2:**
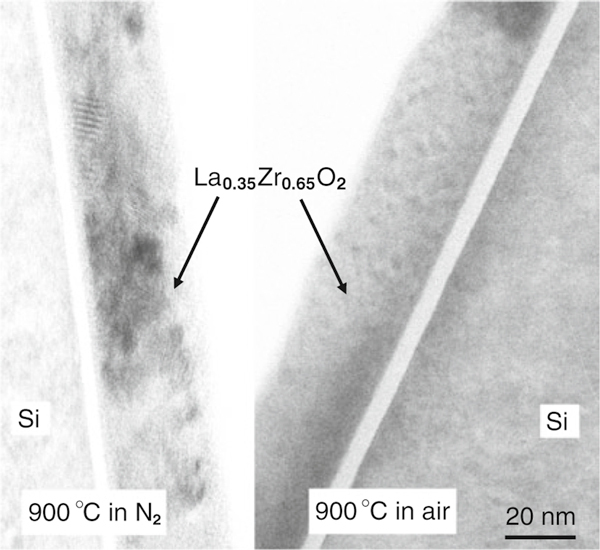
**XTEM images from La_0.35_Zr_0.65_O_2_ samples, which were annealed in air and N_2_ at 900°C for 15 min, respectively**.

XTEM was carried out on both the 900°C PDA samples using a JEOL 2000FX operated at 200 kV. XTEM images in Figure 2 show that equiaxed nano-crystallites of ~4 nm diameter were formed in the air-annealed sample, in comparison with larger ~15-nm crystals for the N_2_-annealed sample. The thickness of the La_0.35_Zr_0.65_O_2_ layers and the IL was also obtained by XTEM. The 35-nm-thick La_0.35_Zr_0.65_O_2_ layers retained their thickness after PDA, but the IL increased from 1.5 nm on the as-deposited samples to 4.5 nm and 6 nm after PDA at 900°C in N_2_ and in air, respectively, which is attributed to either an internal or external oxidation mechanism. Previous medium energy ion scattering (MEIS) results [16] showed the incorporation of some La in the IL, which is reported to increase the k-value of the IL from 3.9 (pure SiO_2_) to ~10 [29].

**Figure 3:**
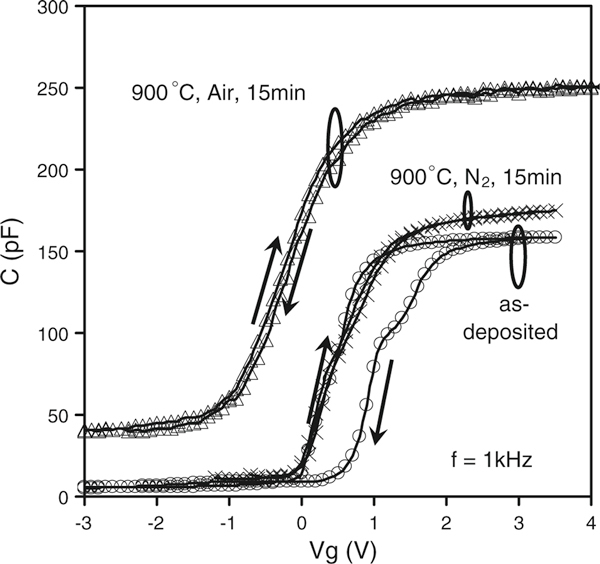
**C–V measurements were carried out at frequency = 1 kHz for as-deposited and PDA samples**.

C–V and C–f measurements were carried out using a HP4192 impedance analyzer and an Agilent E4980A LCR meter at various frequencies (20 Hz–13 MHz) in parallel mode. C–f measurements were performed at a strong accumulation region (Vg = + 3 V). C–V measurements were carried out from strong inversion toward strong accumulation and vice versa. Three typical sets of C–V curves of the as-deposited and PDA samples were shown in Figure 3. PDA was found to significantly reduce the hysteresis to ~10 mV (counterclockwise), independent of the annealing ambient. PDA in air caused a negative shift of the C–V curves due to positive charge generation and also caused an enhanced accumulation capacitance, which originated from a k-value increase in the La_0.35_Zr_0.65_O_2_ layer. Positive charge generation will be discussed first, and then the k-value increase.

From the early days of silicon technology, thermal oxidation of Si has been known to introduce fixed positive charge at the Si/SiO_2_ interface [30]. Positive charge generation during high-temperature processing is not new to thin film SiO_2_ physics; its presence has been detected ever since the pioneering era of Si oxidation in the form of fixed oxide charge that often develops during the oxidation process [31]. The presence of positively charged, over-coordinated oxygen centers in SiO_2_ has been suggested previously in the work of Snyder and Fowler [32]. They showed that the positive charge involved with the *E'* oxygen-vacancy center is in fact associated with over-coordination of an O. Warren et al. suggested that the formation of positively charged over-coordinated O defects is near the Si/SiO_2_ interface [33,34]. The effect of post-deposition oxidation of SiO*x*/ZrO_2_ gate dielectric stacks at different temperatures (500–700°C) on the density of fixed charge was proposed by Houssa et al. [35]. They indicated that increasing oxidation temperature, the density of negative fixed charge is reduced. The net positive charge observed after oxidation at >500°C resembles the charge generated at the Si/SiO_2_ interface by hydrogen in the same temperatures range. They proposed that the observed oxidation-induced positive charge in the SiO*x*/ZrO_2_ gate stack may be related to over-coordinated oxygen centers induced by hydrogen. This also matches our previous observations at the Si/SiO_2_ and Si/SiO_2_/HfO_2_ structures [36,37].

**Figure 4:**
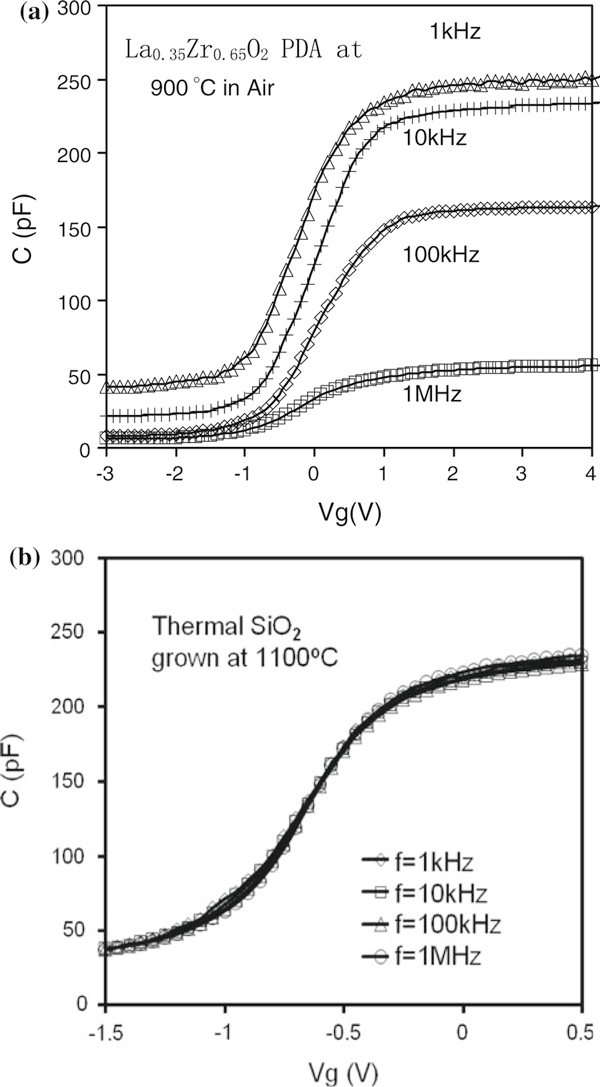
**(a) C–V results at different frequencies from the air-annealed sample**. Significant frequency dispersion was observed. (b) No frequency dispersion in C–V measurements was observed in the thermal oxide (SiO_2_) sample with the back-side contact prepared in the same way as for the LaZrO sample shown in (a).

Before discussing the k-value increase, the causes of frequency dispersion must be totally understood. Figure 4 (a) indicates that a large frequency dispersion was observed during C–V measurements in the air-annealed sample. There are five reasons that may cause the frequency dispersion observed: (1) series resistances, (2) parasitic effects (including back contact imperfection and cables and connections), (3) leakage currents, (4) the interlayer between La_0.35_Zr_0.65_O_2_ layer and semiconductor silicon substrate, or (5) a *k*-value dependence on frequency of the La_0.35_Zr_0.65_O_2_ dielectric. To obtain the genuine intrinsic properties and permittivity of the La_0.35_Zr_0.65_O_2_ dielectric from the CV measurements, the first four effects must be eliminated.

The effects of series resistances and parasitic effects were reported in our previous work [38]. To minimize the effects of series resistances and back contact imperfections (including contact resistance R, contact capacitance C, or parasitic R–C coupled in series, etc.), aluminum back contacts were deposited over a large area of the substrate wafer that was cleaned with a buffered HF solution before aluminum contacts were formed. The same procedure was carried out for all as-deposited, N_2_-annealed, and air-annealed samples. All samples tested had the same or very similar substrate area (~ 2 × 2 cm^2^) to ensure that the effects of series resistance and back contact imperfections were the same for all samples. Furthermore, measurement cables and connections were kept short to further minimize parasitic capacitance effects and were the same for all samples. To provide a comparison with Figure 4a, a C–V measurement on a thermal SiO_2_ sample with the same HF treatment and Al deposition on its back was carried out from the same test system; the results are shown in Figure 4b. It is clear that no frequency dispersion was observed on the thermal SiO_2_ sample. Therefore, the effects of series resistances and parasitic effects are negligible.

**Figure 5:**
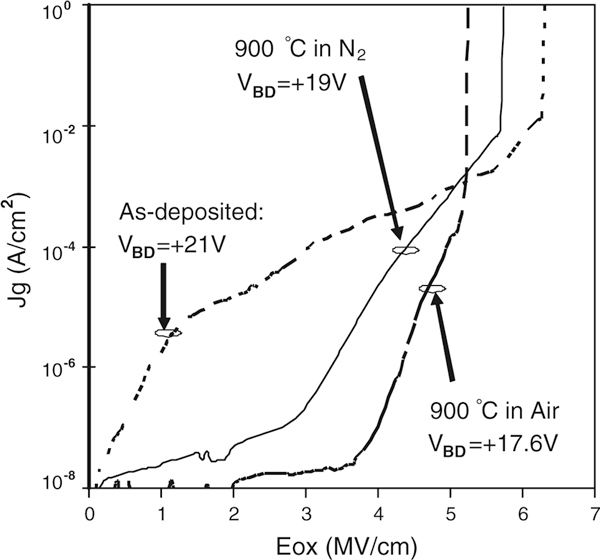
**The relationship between leakage current density (*Jg*) and electric field (*E*_*ox*_) applied across the La_0.35_Zr_0.65_O_2_/IL (IL stands for interfacial layer) stacks for as-deposited and PDA samples**. Break-down voltages (V_BD_) were indicated.

The leakage current characteristics of the La-doped films were evaluated from the I–V measurements, as shown in Figure 5. At low oxide fields (E_ox_ at 0 to +2MV/cm), the leakage current density is improved under positive gate biases after annealing, which is attributed to the thicker IL. However, PDA also causes crystallization that introduces leakage current paths and reduces the break-down voltage. The leakage current densities at +2MV/cm are 1.6 × 10^-5^ Acm^-2^ for as-deposited samples, but below 5 × 10^-8^ Acm^-2^ after the 900°C PDA either in N_2_ or in air. This suggests that the effect of leakage currents on frequency dispersion is negligible during C–V measurements.

Before k-value of the La_0.35_Zr_0.65_O_2_ dielectric is extracted from the strong accumulation capacitance at +3 V (<+1MV/cm), the effect of the presence of the lossy interlayer must be taken into account. The effect was also reported in our previous work [38].

**Figure 6:**
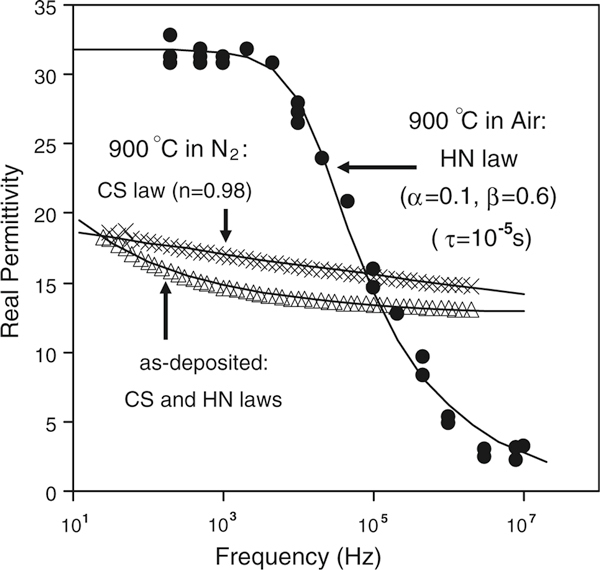
**Frequency dependence of k-value of La_0.35_Zr_0.65_O_2_ dielectric for as-deposited and PDA samples**. Significant dielectric relaxation was observed in the air-annealed sample. Solid lines are the fitting results using equations (1) and (2).

The relationship between the extracted k-value and test frequency shown in Figure 6 indicates that significant dielectric relaxation only occurs in the air-annealed sample. Parasitic effects could not be the cause of the frequency dispersion observed because of the sample preparation and measurement procedures described earlier. Significant frequency dispersion was not seen in other MOSCs fabricated using the same substrates prepared and measured in exactly the same way. We conclude therefore that the frequency dispersion observed in the La_0.35_Zr_0.65_O_2_ film annealed in air is a real material property of this dielectric. There are two important observations in Figure 6: (1) PDA in air increases the k-value of the La_0.35_Zr_0.65_O_2_ dielectric significantly (*k*-value reaches 32 at 1 kHz), along with a significant dielectric relaxation. (2) There is less of an effect on the k-value for the film annealed in N_2_, with a small increase in k-value at some frequencies and a flatter frequency response compared to the as-deposited sample. Both effects of temperature/ambient and causes of dielectric relaxation are discussed later.

Annealing at a high temperature is employed to induce the t- and c-phases in the La-doped ZrO_2_ dielectric from the amorphous samples [15,16]. The addition of La is to stabilize these phases, and the stabilized tetragonal/cubic ZrO_2_ phase gives a higher *k*-value [7-14]. Annealing temperature was reported to range from 400 to 1,050°C, depending on the deposition conditions and substrates of high-k dielectrics that determine the microstructure of the as-deposited samples. It was reported that the germanium substrate requires lower annealing temperatures ranging from 400 to 600°C [7-11]. If the microstructure of the as-deposited LaZrO_2_ samples had already been tetragonal/cubic, annealing at high temperatures would not be necessary [9].

It has been shown previously that dielectric relaxation in the time domain can be described by a power-law time dependence, *t*^-*n *^[26,27], or a stretched exponential time dependence, exp[-(*t/t*_0_)^*m*^] [39,40], where *n* and *m* are parameters ranging between 0 and 1, and *t*_0_ is a characteristic relaxation time.

In the frequency domain, after a Fourier transform, the corresponding dielectric response of *t*^-*n*^ dependence is well described in terms of Curie–von Schweidler (CS) behavior [24,26,27], while the Fourier transform of exp[-(*t/t*_0_)^*m*^] function into frequency domain can be approximated by a Havriliak–Negami (HN) relationship [25], after a great deal of work [41-43]. The CS law and HN relationship can be, respectively, expressed as

(1) $$\varepsilon_{CS}(\omega )-\varepsilon_{\infty }=A{\left(i\omega \right)}^{n-1}$$

(2) $$\varepsilon_{HN}(\omega )-\varepsilon_{\infty }=(\varepsilon_{s}-\varepsilon_{\infty })/{\left[1+{\left(i\omega \tau \right)}^{1-\alpha }\right]}^{\beta }$$

where *ε*_*s*_ and *ε*_*∞*_, are the static and high-frequency limit permittivities, respectively; *τ* is the HN relaxation time; *ω* = 2π*f* is the angular frequency; and *n, α*, and *β* are the relaxation parameters.

A theoretical description of the slow relaxation in complex condensed systems is still a topic of active research despite the great effort made in recent years. There exist two alternative approaches to the interpretation of dielectric relaxation: the parallel and series models [44]. The parallel model represents the classical relaxation of a large assembly of individual relaxing entities such as dipoles, each of which relaxes with an exponential probability in time but has a different relaxation time *t*_*k*_. The total relaxation process corresponds to a summation over the available modes *k*, given a frequency domain response function, which can be approximated by the HN relationship.

The alternative approach is the series model, which can be used to describe briefly the origins of the CS law (the *t*^-n^ behavior). Consider a system divided into two interacting sub-systems [45]. The first of these responds rapidly to a stimulus generating a change in the interaction which, in turn, causes a much slower response of the second sub-system. The state of the total system then corresponds to the excited first system together with the unresponded second system and can be considered as a transient or metastable state, which slowly decays as the second system responds.

In some complex condensed systems, neither the pure parallel nor the pure series approach is accepted and instead interpolates smoothly between these extremes [46]. The CS behavior has to be faster than the HN function at short times and slower than the HN function at long times.

Based on the discussion above, the dielectric relaxation results (shown in Figure 6) have been modeled with the CS and/or HN relationships (see solid lines in Figure 6). The relaxation of the as-deposited film obeyed a mixed CS and HN relationships. After the 900°C PDA, the relaxation behavior of the N_2_-annealed film was dominated by the CS law, whereas the air-annealed film was predominantly modeled by the HN relationship that was accompanied by a sharp drop in the k-value.

Although the exact microstructural cause of these relaxation processes is not clearly known, several mechanisms for the dielectric relaxation have been proposed, including distribution of relaxation time [47], distribution of hopping probabilities [48], space charge trapping [49], self-similar multi-well potential for ionic configurations [45], or double potential well occupied by one electron [50]. However, it has been reported that a decrease in crystal grain size can cause an increase in the dielectric relaxation in ferroelectric relaxor ceramics [51,52]. This relaxation effect has been attributed to higher stresses in the smaller grains [51]. A similar effect appears to have occurred with these La-doped dielectric films, with the 900°C air anneal producing 4-nm diameter equiaxed nano-crystallites within the film, and suffering from a severe dielectric relaxation. The 900°C N_2_-annealed film contains much larger ~15-nm crystals and does not suffer from severe dielectric relaxation. Therefore, the physical processes behind the relaxation are probably related to the size of the crystal grains formed during annealing.

## Conclusions

PDA at 900°C either in N_2_ or in air causes crystallization (t- or c-phases) of the La_0.35_Zr_0.65_O_2_ dielectric. Larger crystal grain sizes were observed in the N_2_-annealed sample than in the air-annealed sample. Following PDA in N_2_, the k-value was maintained and the dielectric relaxation was reduced. However, PDA in air causes a significant increase in k-value (32 at 1 kHz) and a significant dielectric relaxation, probably associated with smaller crystal grain sizes. The relaxation behavior of the as-deposited sample can be modeled using the mixed CS and HN relationships. PDA in N_2_ suppressed the HN law, while the CS law was removed following PDA in air.
